# Molecular Mechanisms of (*R*,*R*)ZX-5 on NO Synthesis and Its Anti-Angiogenic Effect

**DOI:** 10.3390/ijms13032717

**Published:** 2012-02-29

**Authors:** Li Pan, Jia-Liang Hu, Wen-Jing Wang, Xiao-Juan Zhang, Jin Wei, Zhen-Dong Liu, Yi-Hua Zhang, Han-Mei Xu

**Affiliations:** 1 State Key Laboratory of Natural Medicines, College of Life Science and Technology, China Pharmaceutical University, Nanjing 210009, China; E-Mails: panli0225@163.com (L.P.); jialiang_hu@yahoo.cn (J.-L.H.); yuejiefanghui@126.com (W.-J.W.); zhangxiaojuan_2010@126.com (X.-J.Z.); 67747374@qq.com (J.W.); liuzhengdong110@yahoo.com.cn (Z.-D.L.); 2 Department of Pharmacy, University of China Pharmaceutical, Nanjing 210009, China

**Keywords:** (*R,R*)ZX-5, age-related macular degeneration, choroidal neovascularization, signal pathways, angiogenesis

## Abstract

(*R*,*R*)ZX-5 is a NO regulatory compound, which could significantly increase choroidal blood flow in New Zealand rabbit. The aim of this paper is to investigate the molecular mechanism of (*R*,*R*)ZX-5 promoting NO production. Besides this, we also investigated the antiangiogenic activity of (*R*,*R*)ZX-5. Analysis of Western blot showed that (*R*,*R*)ZX-5 up-regulated the expression of Akt, *p*-Akt (Thr473), eNOS and *p*-eNOS (Ser1177), down-regulated the expression of Cyclin D1 in human retinal endothelial cells and escalated the intracellular free Ca^2+^ concentration. Additionally, (*R*,*R*)ZX-5 inhibited the growth of blood vessels in the chick chorioallantoic membrane model. It is concluded that (*R*,*R*)ZX-5 promotes choroidal blood flow through PI3K/Akt-eNOS and Akt-Ca^2+^-eNOS pathways. Additionally, (*R*,*R*)ZX-5 can inhibit angiogenesis.

## 1. Introduction

Age-related macular degeneration (AMD) is an eye condition that leads to the deterioration of the center of the retina, called the macula, leading to loss of central vision. AMD is the fastest growing form of macular degeneration, and is the number one cause of severe vision loss and legal blindness in adults over 60 years of age in the U.S. There are two types of AMD—“dry” and “wet”. During the development of dry AMD, which is also known as non-neovascular AMD, cellular debris called drusen accumulates, and retinal pigment epithelial and choriocapillary atrophy. Wet AMD, also known as neovascular AMD, is more severe than dry AMD. Choroidal neovascularization (CNV) is the creation of new blood vessels in the choroids layer of the eye. During the process of Wet AMD, blood vessels grow from the choroid behind the retina. Wet AMD affects 10% of all patients, and accounts for more than 80% of vision loss in AMD [1, 2]. Decrease of the choroidal circulation is considered to be one of the reasons for CNV. It is important to improve and promote choroidal blood flow to alleviate AMD syndromes [3, 4].

Nitric oxide (NO) production by vascular endothelium is important in the regulation of blood flow. Reduced production of NO can adversely affect blood flow and ither vascular functions. NO is a key signaling messenger, which is synthesized in mammalian tissues by nitric oxide synthase (NOS). Endothelial nitric oxide synthase (eNOS) is the predominant NOS in vascular endothelial cells. Once NO is formed by eNOS, it plays an important role in blood flow and other vascular functions [3]. Moreover, pathological studies found that expression of eNOS was reduced in the choroidal blood vessels and choroidal vascular endothelial cells in patients with AMD [5]. Evidence indicated that the reduction of eNOS expression and nitric oxide (NO) production might contribute to the formation of AMD.

In view of this, we designed and synthesized more than 200 NO regulatory compounds. These compounds were screened by Prof. George C. Y. Chiou [4] at Texas A & M University (Texas, USA) and found that compound (*R*,*R*)ZX-5 could significantly increase choroidal blood flow in New Zealand rabbit, but did not affect the blood flow in iris and ciliary body. (*R*,*R*)ZX-5 is the *cis*-1-phenyl-3-(3-methoxy-2-propoxy-5-[4-(3,4,5-trimethoxyphenyl)-1,3-dioxolane-2-yl]phenyl) thiourea. Moreover, the compound’s trans-isomer (*S*,*S*)ZX-5 is not able to promote choroidal blood flow in New Zealand white rabbit [4], suggesting that the promotion of choroidal blood flow is specific to *cis*-isomer (*R*,*R*)ZX-5.

(*R*,*R*)ZX-5 has been proven to improve the choroidal blood flow without affecting the sclera and ciliary bodies in New Zealand white rabbits via increasing NO production [6]. In order to clarify the molecular mechanism of (*R*,*R*)ZX-5 promoting NO production, we investigated and found that the compound can increase the expression of eNOS and NO release in human umbilical vein endothelial cells [7]. Previous studies found that in addition to MAPK Kinase/ERKs, eNOS activity was also regulated by phosphoinositide 3-kinase/Akt and Ca^2+^/Calm. So we investigated to see if ZX-5 increases NO release through PI3K/Akt/eNOS/NO pathway or PI3K/Akt/Ca^2+^/eNOS/NO pathway.

In addition, inhibiting choroidal neovascularization (CNV) is an important treatment of AMD. So we studied the antiangiogenic activity of ZX-5. A useful model for studying angiogenesis and anti-angiogenesis *in vivo* is the chorioallantoic membrane (CAM) of the chicken embryo [2, 8]. The main advantages of CAM angiogenesis assays are their low cost, simplicity, reproducibility, and reliability [9].

## 2. Results and Discussion

HREC was used in the current research for a few reasons; first, HRECs and HCECs (human choroidal endothelial cells) are both positive for the cell markers VEGFR1, VEGFR2, CD31, CD34 and von Willebrand’s factor (vWF) [10]. Second, VEGF isoforms 121 and 165 were found to be equally potent in stimulating endothelial cell proliferation. And third, the anti-VEGF treatments ranibizumab and bevacizumab were effective in decreasing proliferation of HCEC and HREC. Finally, the results in this work indicated that (*R*,*R*)ZX-5 may also be applied in such animal models as proliferative diabetic retinopathy.

### 2.1. Effect of ZX-5 on Akt, Phospho-Akt (Thr473), eNOS and Phospho-eNOS (Ser1177) Levels in HRECs

**Figure 1:**
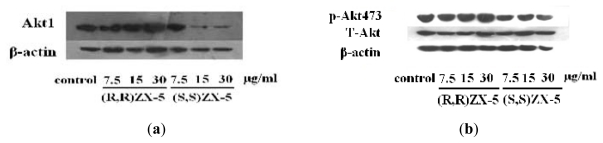
Western blot analysis of Akt and *p*-Akt (Thr473) expression in HRECs after treatment. **a**, **b**: Lane 1, DMSO; Lane 2–4, (*R*,*R*)ZX-5 treatment at a dose of 7.5, 15, 30 μg/mL; Lane 5–7, (*S*,*S*)ZX-5 treatment at a dose of 7.5, 15, 30 μg/mL (β-actin as the control). *p*-Akt473, Akt phosphorylation at threonine 473; T-Akt, total Akt.

**Figure 2:**
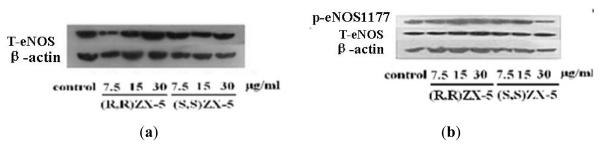
Western blot analysis of eNOS and *p*-eNOS (ser1177) expression in HRECs after treatment. **a**, **b**: Lane 1, DMSO; Lane 2–4, treatment with (*R*,*R*)ZX-5 at a dose of 7.5, 15, 30 μg/mL; Lane 5–7, treatment with (*S*,*S*)ZX-5 at a dose of 7.5, 15, 30 μg/mL (T-eNOS as the control).

To demonstrate the effect of ZX-5 on the PI3K/Akt pathway in HRECs, the expression of Akt, Phospho-Akt (Thr473), eNOS and Phospho-eNOS (Ser1177) were tested. Compared with DMSO-treated control cells, the protein expression level of Akt, Phospho-Akt (Thr473), eNOS and Phospho-eNOS (Ser1177) were up-regulated in a concentration dependent manner in (*R*,*R*)ZX-5-treated cells, whereas (*S*,*S*)ZX-5 down-regulated the expression of Akt and had no influence on the expression of phospho-Akt (Thr473), eNOS and *p*-eNOS (Ser1177) (Figures 1 and 2).

PI3K/Akt signaling pathway is one of the important intracellular signal transduction pathways in response to the extracellular signals. The key effector molecule Akt in this signaling pathway exerts its effects in the cell by phosphorylating a variety of downstream substrates. The downstream targets of Akt include BAD, Caspase 9, FKHR, GLUTs, Ca^2+^ and eNOS [11]. eNOS phosphorylation at Ser1177 and Ser (Thr) 613 can enhance its activity which is affected by the PI3K/Akt.

**Figure 3:**
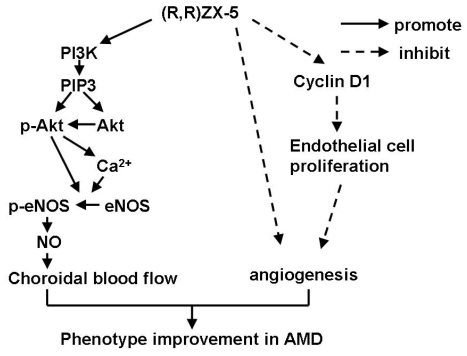
(*R*,*R*)ZX-5 promotes choroidal blood flow through PI3K/Akt and Akt-Ca^2+^-eNOS pathway and inhibits CNV through inhibiting angiogenesis.

We think that (*R*,*R*)ZX-5 up-regulates eNOS expression and increases NO release through the activation of PI3K/Akt-eNOS pathway [12]. The signal pathway transduction are as follows [13]: First, PI3K is activated, which generates phosphatidylinositol-3,4,5-triphosphate (PI3P) and phosphatidylinositol-3,4-diphosphate (PI2P). PI3P recruits PDK1 and Akt serine/threonine kinase at the plasma membrane, which results in the phosphorylation and activation of Akt. Then *p*-Akt is released from cellular membranes and makes eNOS phosphorylated at Ser1177 and Ser (Thr) 613. Further, *p*-eNOS catalytes l-arginine to generate NO (Figure 3).

### 2.2. Effect of ZX-5 on Intracellular Free Ca^2+^ Concentration in HRECs

**Figure 4:**
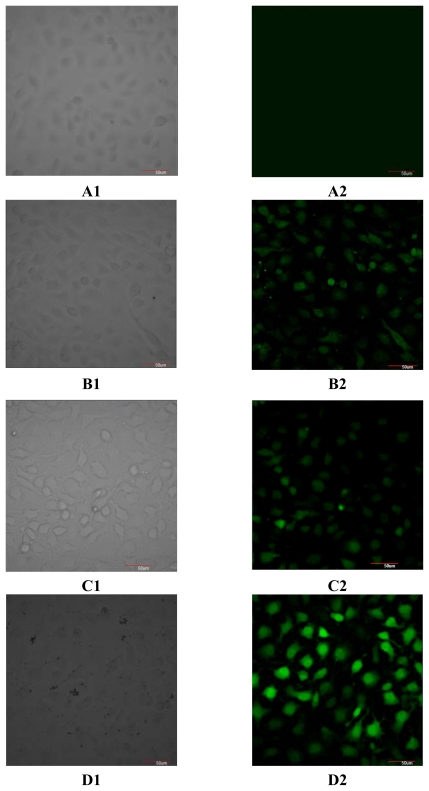
Cell loading fluo-3 in Laser scanning confocal microscope. **A1**, **A2**: HRECs in the absence of fluo-3 and ZX-5 treatment. **B1**, **B2**: HRECs with fluo-3 and without ZX-5 treatment. **C1**, **C2**: HRECs in the presence of fluo-3 after (*S*,*S*)ZX-5 treatment, at a dose of (*S*,*S*)ZX-5 is 30 μg/mL. **D1**, **D2**: HRECs in the presence of fluo-3 after (*R*,*R*)ZX-5 treatment, at a dose of (*R*,*R*)ZX-5 is 30 μg/mL. A1, B1, C1, D1: at visible light; A2, B2, C2, D2: fluorescence.

The intracellular free Ca^2+^ concentration in the ZX-5 treated HRECs was detected. As (*R*,*R*)ZX-5 up-regulated the protein expression level of Akt, Phospho-Akt (Thr473), eNOS and Phospho-eNOS (Ser1177) in a concentration range of 7.5 to 30 μg/mL, a concentration of 30 μg/mL was used in this experiment. The intracellular free Ca^2+^ concentration was escalated significantly by (*R*,*R*)ZX-5 treatment, but (*S*,*S*)ZX-5 did not change the intracellular free Ca^2+^ concentration (Figure 4).

Calcium is an important and ubiquitous intracellular messenger, controlling a diverse range of cellular processes, such as gene transcription, muscle contraction and cell proliferation [14]. In response to adequate stimuli, [Ca^2+^]i (Intracellular Ca^2+^ concentration) increases, oscillates and decreases, leading to the activation, modulation and termination of cell function. Numerous channels and pumps allow this particular cation to enter and exit cells and move between the cytosol and intracellular stores. Activation of NOS requires [Ca^2+^], calmodulin, calcium-activated phosphatase, NADPH, and glutamate. eNOS (endothelial NOS) is constitutively expressed as latent enzymes and require a higher concentration of Ca^2+^ for the enzyme activity [15].

We think that after *p*-Akt is released from cellular membranes and makes eNOS phosphorylated at Ser1177 and Ser (Thr) 613, *p*-Akt also increases [Ca^2+^]i, which enhances the activity of eNOS further. In this way, *p*-eNOS catalytes l-arginine to generate more NO (Figure 3).

### 2.3. Effect of ZX-5 on Cyclin D1 Level in HRECs

**Figure 5:**
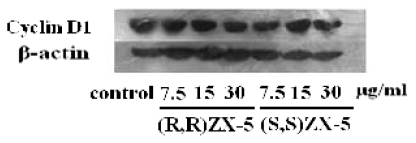
Western blot analysis of Cyclin D1 expression in HRECs after treatment. Lane 1, DMSO; Lane 2–4, (*R*,*R*)ZX-5 treatment at a dose of 7.5, 15, 30 μg/mL; Lane 5–7, (*S*,*S*)ZX-5 treatment at a dose of 7.5, 15, 30 μg/mL (β-actin as the control).

To demonstrate the effect of ZX-5 on Cyclin D1 in HRECs, the expression of Cyclin D1 was assessed. Compared with DMSO-treated control cells, the protein expression level of Cyclin D1 was down-regulated in a concentration dependent manner in (*R*,*R*)ZX-5-treated cells, and (*S*,*S*)ZX-5 also down-regulated the expression of Cyclin D1 (Figure 5).

### 2.4. Inhibitory Effects of (R,R)ZX-5 on Angiogenesis in the CAM Model

**Figure 6:**
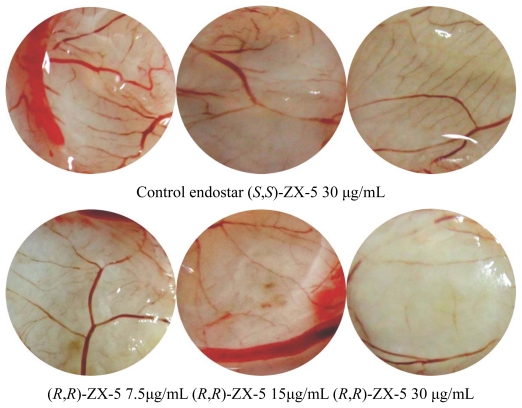
Anti-angiogenic effect of ZX-5 on CAM. CAMs were treated with 0.1% DMSO, (*R*,*R*)-ZX-5 7.5 μg/mL, 15 μg/mL, 30 μg/mL, (*S*,*S*)-ZX-5 30 μg/mL, endostar for 48 h.

**Figure 7:**
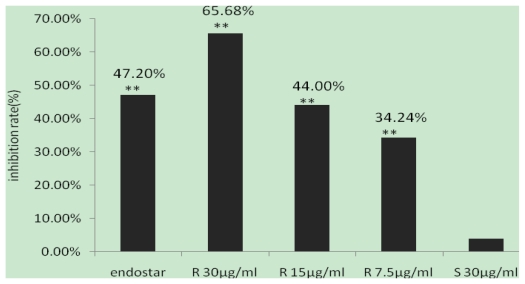
Inhibition rate of angiogenesis chick chorioallantonic membrane. The chick chorilallantonic membrane was treated with endostar(5 mg/mL), (*R*,*R*)-ZX-5(at a dose of ZX-5 was 30 μg/mL, 15 μg/mL, 7.5 μg/mL, respectively), (*S*,*S*)-ZX-5 (30 μg/mL), and physiological saline (0.1% DMSO). The data were presented as mean ± SD, ** *p* < 0.01 (*n* = 5, five sections in each egg).

A CAM model was setup to evaluate the effect of (*R*,*R*)ZX-5 on angiogenesis. 48 h after drug administration into the chick embryos, medium and small blood vessels were counted within the 15 mm radius from the center of the drug carrier plate. The effect of ZX-5 on angiogenesis has been shown in Figure 6. Compared with negative control groups, the angiogenesis were inhibited by (*R*,*R*)ZX-5 (inhibition ratios were 34.24%, 44.00% and 65.68% at 7.5, 15 and 30 μg/mL, respectively) in a concentration dependent manner. (*S*,*S*)ZX-5 had no effect on the angiogenesis (inhibition ratio was 3.76% at 30 μg/mL) (Figure 7).

Angiogenesis is a complex process, usually including vascular basement membrane degradation, endothelial cell activation, proliferation, migration, re-formation of new blood vessels and vascular networks. Cyclin D1 is cell cycle-related protein interacting with multiple other proteins which acts on G1 phase and promotes cells to enter S phase. We tested the effect of ZX-5 on Cyclin D1 expression on protein level in HREC. It was found that (*R*,*R*)ZX-5 can significantly inhibit angiogenesis and down-regulate the expression of Cyclin D1. Downregulation of Cyclin D1 expression may partially contribute to (*R*,*R*)ZX-5’s anti-angiogenic effect. (*S*,*S*)ZX-5 also down-regulated Cyclin D1 expression, but it had no influence on angiogenesis.

## 3. Experimental Section

### 3.1. Cell Culture

The human retinal endothelial cells line (HRECs) was purchased from the American Type Culture (ATCC, Shanghai, China) and maintained in T75 flasks at 37 °C in an atmosphere of 5% CO_2_. Cells were maintained in Dulbecco’s modified Eagle’s medium (DMEM) (Invitrogen, USA) supplemented with 10% fetal bovine serum (FBS), and antibiotics (penicillin G 100 U/mL, streptomycin sulfate; Generay Biotech Co., Ltd., Shanghai, China). The cells were allowed to reach confluence and then subcultured in 6-well plates at 8.0 × 10^5^ cells/well until confluent. To test the expression level of each protein, prepared cells were starved for 24 h in serum-free media before assay.

### 3.2. Cell Treatment

(*R*,*R*)ZX-5 and (*S*,*S*)ZX-5 were dissolved in Dimethyl sulfoxide (DMSO).

HRECs incubated in DMEM medium were divided into 3 groups: group 1 treated with DMSO in serum-free media, group 2 treated with (*R*,*R*)ZX-5 (7.5, 15, 30 μg/mL) in serum-free media, group 3 treated with (*S*,*S*)ZX-5 (7.5, 15, 30 μg/mL) in serum-free media. All cells were incubated for 24 h before western blot assay.

### 3.3. Western Blot Analysis

HRECs were seeded in 6-well culture plates with 8.0 × 10^5^ cells per well and incubated for 24 h. Then the cells were treated by (*R*,*R*)ZX-5 or (*S*,*S*)ZX-5 for 24 h. The cells were harvested with 0.05% trypsin/0.53 mM EDTA (Hyclone, UT, USA), washed with phosphate-buffered saline (PBS), and resuspended in 100 μL of all Mammalian protein Extraction Reagent (Generay Biotech Co., Ltd. Shanghai, China). Concentrated proteins were separated on a 12% gel for the detection of Cyclin D1, Akt, *p*-Akt (Thr473), a 8% gel for the detection of eNOS, *p*-eNOS (Ser1177), respectively. The separated proteins on the gel were transferred to PVDF membrane. Then the PVDF membrane (Millipore, MIT, USA) was incubated with a 1:1000 dilution of Cyclin D1, Akt, *p*-Akt (Thr473), eNOS, *p*-eNOS (Ser1177) and β-actin (Santa Cruz biotechnology, USA) antibodies. After incubation with the appropriate secondary antibodies (Multi Sciences Biotech Co., Ltd., Hang Zhou, China), blots were treated with the ECL reagents (Beyetime, Jiangsu province, China) and exposed to photographic film to detect protein expression.

### 3.4. Measurement of Intracellular Free Ca^2+^ Concentration by LSCM (Laser Scanning Confocal Microscope)

Fluo-3/AM (Multi Sciences Biotech Co., Ltd., Hang Zhou, China) was used as an intracellular free Ca^2+^ fluorescent probe. In short, after the treatment with 30 μg/mL (*R*,*R*)ZX-5 or (*S*,*S*)ZX-5 respectively, cells were incubated with 3 μg Fluo-3/AM for 30 min at 37 °C (the final concentration of extracellular Fluo-3/AM was 3 μg/mL). Then the cells were washed 3 times with D-Hank’s solution to remove the extracellular Fluo-3/AM and the intracellular fluorescence was detected using a LSCM (Olympus Fv-1000, Olympus Corporation, Japan) with the excited light at a wavelength of 488 nm and the emission light at 525 nm.

### 3.5. CAM Angiogenesis Assays

The eggs which have been fertilized 6 days were used for the experiment and were incubated at 70% of humidity and 37 °C. A window was opened on the top of each egg after 1 day incubation. The windows were covered with sterile tape and the eggs were returned to the incubator. After another 1 day, the transparent tape was cut open and a drug carrier plate with a 15 mm diameter was placed in the chamber. Twenty microliters of each test reagent was added onto the plate. Test substances including (*R*,*R*)ZX-5 (the dose of ZX-5 was 7.5 μg/mL, 15 μg/mL, 30 μg/mL, respectively) and (*S*,*S*)ZX-5 (30 μg/mL). The physiological saline (0.1% DMSO) and endostatin (5 mg/mL) were used as negative and positive controls, respectively. Forty-eight hours after implantation of the filter paper disc, the pseudo-air chamber of chick embryos were opened and fixed in the fixative (formaldehyde-acetone, 1:1) for 5 min. The chick embryo was subsequently removed, and the CAM was carefully peeled off from the egg shell and laid out flat for photography. The number of medium-small blood vessels within a radius of 15 mm from the center of the carrier plate was counted.

### 3.6. Statistical Analysis

Data were presented as the mean ± SD. The statistical significance of the differences was determined by the method of analysis of variance (ANOVA) or unpaired two-tailed *t*-tests; a value of *p* < 0.05 was considered significant.

## 4. Conclusions

Previous research confirmed that (*R*,*R*)ZX-5 can improve choroidal blood flow via increasing NO production (4, 6). These effects were based on the up-regulation of eNOS expression through PI_3_K/Akt and Akt-Ca^2+^-eNOS pathways. (*R*,*R*)ZX-5 also showed anti-angiogneic effect in a CAM assay. (*R*,*R*)ZX-5 is a promising candidate worthy of further investigation.
